# Zein Nanoparticles Loaded with *Vitis vinifera* L. Grape Pomace Extract: Synthesis and Characterization

**DOI:** 10.3390/nano15070539

**Published:** 2025-04-02

**Authors:** Ana Guadalupe Luque-Alcaraz, Jesús Antonio Maldonado-Arriola, Pedro Amado Hernández-Abril, Mario Enrique Álvarez-Ramos, Cynthia Nazareth Hernández-Téllez

**Affiliations:** 1 Department of Biomedical Engineering, Universidad Estatal de Sonora, Hermosillo 83100, Mexico; ana.luque@ues.mx (A.G.L.-A.); jesusantonio.maldonado@unison.mx (J.A.M.-A.); pedro.hernandez@ues.mx (P.A.H.-A.); 2 Department of Industrial Engineering, Universidad de Sonora, Hermosillo 83000, Mexico; 3 Department of Physics, Universidad de Sonora, Hermosillo 83000, Mexico

**Keywords:** nanoparticles, zein, grape pomace, extract, polyphenols

## Abstract

This study investigates the synthesis and characterization of zein nanoparticles (ZNp) loaded with grape pomace extract (GPE) from *Vitis vinifera* L. for applications in controlled release and antioxidant delivery. Grape pomace, a byproduct of the winemaking industry, is rich in bioactive compounds, including phenols and flavonoids, which possess antioxidant properties. To overcome the limitations of these compounds, such as photosensitivity and thermal degradation, they were incorporated into zein nanoparticles using the antisolvent technique. The physicochemical properties of the ZNp-GPE system were thoroughly characterized, including size, morphology, ζ-potential, and total phenol content. Results showed high encapsulation efficiency (89–97%) and favorable loading capacities. Characterization techniques, such as scanning electron microscopy (SEM), Fourier transform infrared spectroscopy (FTIR), and dynamic light scattering (DLS), confirmed that GPE was successfully incorporated into the nanoparticles, thereby enhancing their antioxidant properties. The encapsulation process did not significantly alter the spherical morphology of the nanoparticles, and loading GPE resulted in a decrease in particle size. Total phenol content analysis showed that the ZNp-GPE nanoparticles effectively retained these compounds, confirming their potential as efficient delivery systems for antioxidants. This approach not only provides a method for protecting and enhancing the bioavailability of natural antioxidants but also contributes to the valorization of agricultural waste, promoting sustainability in bio-based industries.

## 1. Introduction

Grape pomace, derived from the common grape vine (*Vitis vinifera* L.), is a by-product of the wine industry obtained during the winemaking process, composed of pulp, skin, and seeds from the grape. Although it has traditionally been considered a residue of little economic value, in recent years, it has gained relevance due to its high content of bioactive compounds, such as phenols, flavonoids, resveratrol, and organic acids, which possess antioxidant and anti-inflammatory properties [1,2]. Various studies have highlighted the potential of these compounds in diverse industrial applications, particularly in sectors such as food technology [3], cosmetics [4], and pharmaceuticals [5].

Grape pomace extract (GPE) has been extensively studied for its ability to neutralize free radicals, making it an excellent candidate for antioxidant applications, particularly in the prevention of cardiovascular and neurodegenerative diseases [6]. However, one of the main challenges for natural extracts is their photosensitivity and thermal degradation, which limits their effectiveness [7]. An effective strategy to overcome these limitations is to incorporate polymeric nanoplatforms, which protect the bioactive compounds from degradation and allow them for controlled release in the organism [8].

Ethanol extraction is a simple and cost-effective method for obtaining bioactive compounds from natural sources. Ethanol serves as a versatile solvent capable of dissolving both polar and, to a certain extent, non-polar compounds, making it a valuable tool for both research and industry. Furthermore, it is a straightforward, accessible, and less harmful technique than other organic solvents, making it a suitable choice for the food, pharmaceutical, and cosmetic industries. Research has demonstrated that this extraction method effectively retrieves polyphenols from agricultural and industrial byproducts, such as plant residues and fruit processing waste. Utilizing these secondary materials fosters a more efficient and sustainable use of natural resources, preventing the loss of bioactive compounds with potential applications.

Due to their small size and high surface-to-volume ratio, polymeric nanoparticles possess exceptional properties that make them ideal for applications in controlled drug and bioactive compound delivery across various sectors, including medicine [9], food [10], and pharmaceuticals [11]. Zein, a plant protein extracted from corn, has been widely used in nanoparticle synthesis due to its biocompatible and biodegradable properties. Zein nanoparticles can encapsulate bioactive compounds such as antioxidants and drugs, protecting them from external factors like light and oxygen and enabling sustained release [12]. Previous research has shown that ZNp can effectively interact with compounds such as resveratrol, found in GPE, facilitating its transport and absorption in the body [13].

Zein nanoparticles have gained attention in biomedical applications due to their biocompatible and biodegradable properties [14]. These nanoparticles are utilized in the controlled release of drugs and bioactive compounds due to their ability to encapsulate active ingredients, such as antioxidants [15], and protect them from degradation caused by environmental factors, including light and oxygen [16]. Their application in the biomedical field encompasses the treatment of cardiovascular and neurodegenerative diseases [17], where antioxidants play a crucial role in preventing cellular damage and inflammation [18]. In addition to these uses, zein nanoparticles show potential in cancer therapy [19], wound healing [20], and tissue regeneration by facilitating the targeted delivery of therapeutic agents and promoting cellular growth [21,22]. The use of zein nanoparticles to enhance the bioactivity and stability of antioxidant compounds, as well as their ability to deliver a wide range of biomolecules, represents a promising strategy in medicine, including drug delivery systems [23], nanocarriers for gene therapy [24], and regenerative medicine [25].

The primary objective of this study is to synthesize ZNp loaded with GPE (ZNp-GPE) using the solute–solvent technique, thereby developing an efficient system for incorporating the bioactive compounds present in GPE, such as flavonoids and resveratrol. The key advantage of the solute–solvent technique lies in its capacity to synthesize nanoparticles with low polydispersity, which is crucial for achieving the controlled release of bioactive compounds. This process enables the modification of the physicochemical properties of the nanoparticles, such as their size and shape, which facilitates their suitability for specific applications in the controlled release of antioxidant compounds [26].

This approach can improve the bioavailability of these compounds and prolong their therapeutic activity. This study conducts a detailed evaluation of the physicochemical properties of the nanoparticles, such as hydrodynamic diameter, ζ-potential, morphology, and size, using techniques such as SEM, FTIR, and DLS. These analyses help to determine the stability and the ability of the nanoparticles to interact with the bioactive compounds.

This study contributes to the development of an innovative strategy for the controlled release of bioactive compounds. Also, it supports the valorization of byproducts from the wine industry, such as grape pomace, which is typically discarded. Utilizing this waste to develop biomedical systems promotes sustainability by reducing waste and utilizing underused resources, contributing to the circular economy, and creating high-value-added products from agro-industrial by-products [27,28,29].

The objective of this study was to evaluate the incorporation of GPE in ZNp to determine its physicochemical properties, such as size, shape, and stability, as well as its potential use as a protective agent for bioactive compounds. The interaction between the extract and the zein nanoparticles could influence both the surface characteristics and the internal structure of the particles, affecting their overall compaction and stability.

## 2. Materials and Methods

### 2.1. Reagents

All reagents and solvents used were analytical grade. The Folin–Ciocalteu phenol reagent was purchased from Hycel (Zapopan, Jal, Mexico), gallic acid was obtained from Jalmeck (San Nicolás de los Garza, NL, Mexico), and zein was sourced from Sigma Aldrich (St. Louis, MO, USA). Other analytical-grade reagents were also used from Sigma Aldrich (St. Louis, MO, USA).

### 2.2. Sample Collection and Preparation

**Figure 1:**
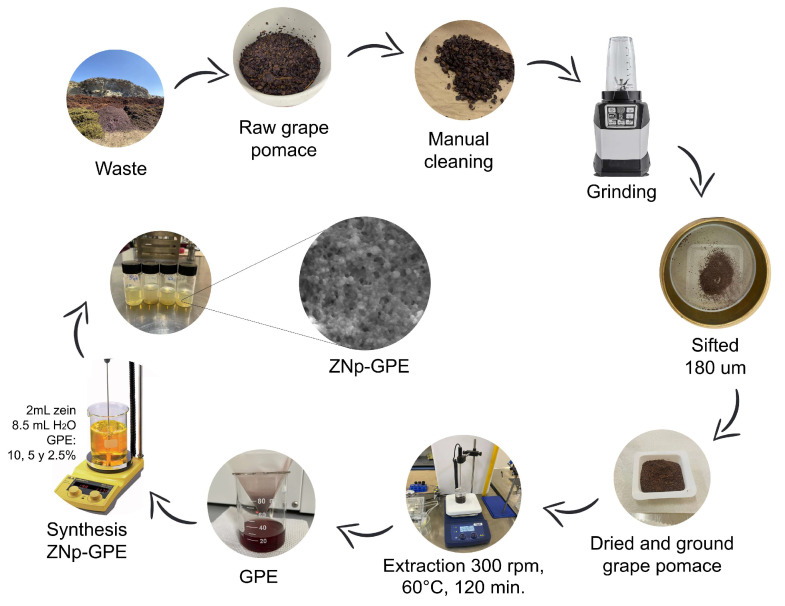
Process of employing grape pomace, extraction of polyphenolic compounds, and synthesis of ZNp loaded with GPE.

In this study, grape pomace (*Vitis vinifera* L.) of the Shiraz variety was used, collected from the L.A. CETTO plant located in Valle de Guadalupe, Ensenada, Mexico. Grape pomace, a byproduct of the winemaking industry, was transported in an icebox to the Universidad Estatal de Sonora (UES) in Hermosillo, Sonora, where it was stored in a freezer at −18 °C until further processing. Before analysis, a manual cleaning process was performed to remove stems, seeds, and leaves, retaining only the grape skin. The skin was then air-dried at room temperature for 48 h and subsequently ground using a 600 W processor. The resulting sample was sieved using an 80-mesh test sieve with 180 µm openings (W.S. Tyler, Mentor, OH, USA), as illustrated in Figure 1. Finally, the sample was stored in darkness at room temperature until its use in the extraction process.

#### Ethanolic Extraction of Grape Pomace Extract (GPE)

The extraction of phenolic compounds was carried out using a solid-liquid extraction process, following the methodology reported by Antoniolli et al. (2015) with some modifications [30]. For this, the ground grape pomace sample was placed in a beaker along with an aliquot of the extraction solvent (ethanol/water, 50:50 *v*/*v*) at a ratio of 25:1 relative to the dry sample mass. The procedure was conducted under continuous agitation at 300 rpm, 60 °C for 120 min. Subsequently, the extract was filtered using filter paper and concentrated in an oven at 40 °C for 24 h (Figure 1). Finally, the concentrated extract was stored under refrigeration for further analysis.

### 2.3. Preparation of Zein-PE-Loaded Nanoparticles (ZNp-GPE)

ZNp and ZNp-GPE were synthesized using the antisolvent precipitation technique [8]. To prepare ZNp, a solution containing 214.7 mg of zein in 15 mL of 90% ethanol was prepared, resulting in a concentration of 14.32 mg/mL of zein. In a separate beaker, 8.5 mL of water (aqueous phase) was added and placed on magnetic stirring. Then, 2.0 mL of the zein solution was gradually added dropwise with a syringe. Lastly, the mixture was stirred for an additional 10 min to achieve a final zein concentration of 2.6 mg/mL. To prepare the ZNp-GPE, the extract was incorporated in the aqueous phase to obtain three different GPE concentrations: 10%, 5.0%, and 2.5% (*v*/*v*), corresponding to samples ZNp-GPE1, ZNp-GPE2, and ZNp-GPE3, respectively (Figure 1).

### 2.4. Characterization

#### 2.4.1. Characterization of the Absorption Spectra

The spectral absorption data for the samples were recorded using a VE-5100UV UV–vis spectrophotometer (VELAB, Tlalpan, CDMX, Mexico). Before measurement, the samples were diluted in ultrapure water at a 1:31 ratio. A quartz cuvette served as the sample holder. To ensure accuracy, a baseline correction was applied by subtracting the water absorption spectrum from the sample readings.

#### 2.4.2. Quantification of Total Phenol Content

The analysis is based on a redox reaction involving the phenolic components of the sample and the Folic–Ciocalteu reagent, which is a mixture of sodium tungstate and sodium molybdate in phosphoric acid, resulting in a yellow phosphomolybdotungstic acid; the phenolic groups reduce this acid and change to an intense blue color dependent on the amount of phenols in the sample. The colored sample exhibits a maximum absorption at 760 nm [31].

In this study, the procedure was performed in triplicate test tubes for each treatment. A total of 900 µL of deionized water and 100 µL of sample were added, followed by 250 µL of Folin–Ciocalteu reagent previously diluted in a 1:2 ratio (reagent/water). The mixture was incubated in darkness for 5 min, after which 1250 µL of 20% Na_2_CO_3_ was added. Finally, the solution was incubated in darkness for 90 min. Spectrophotometric measurements were conducted at 760 nm using a spectrophotometer [32]. For quantification, a standard curve (R^2^ = 0.9997) was previously established using gallic acid. The results were expressed as µmol of gallic acid equivalents per gram of dry weight (µmol GAE/g dw) using the following equation:

$$C=\frac{C1\cdot V}{m}$$

where *C* represents the total phenols, gallic acid equivalent in milligrams per gram (mg GAE/g), *C*1 is the concentration of gallic acid in milligrams per milliliter (mg GAE/mL), *V* is the volume of the sample in milliliters (mL), and *m* is the dry sample weight in grams (g).

#### 2.4.3. Nanoparticles Entrapment Capacity

The entrapment capacity of ZNp-GPE1, ZNp-GPE2, and ZNp-GPE3 was evaluated. Preparation was performed as described in the previous section. The procedure followed to calculate the total phenolic content of the extract was used to determine the loading capacity, as reported in other studies with minor modifications [33]. The ZNp-GPE were separated from the suspension by using a multiple centrifuge 120V FC5718 (OHAUS, Miguel Hidalgo, CDMX, Mexico) at 7500 RPM and 4 °C for 20 min. The trapped amount of GPE within the nanoparticles was calculated with the difference between the total GPE added to the formation medium and the unbound GPE leftover as supernatant, which was measured by UV–vis spectrophotometry at 760 nm using a VE-5100UV UV–vis spectrophotometer (VELAB, Tlalpan, CDMX, Mexico). A standard curve of gallic acid was prepared to determine the total amount of extracted phenols indirectly. A mixture of solvents was used and measured as a baseline to be subtracted from the sample data. The association efficiency (AE) and the loading efficiency (LE) of the GPE were calculated with the following equations [34]:

$$Association\;efficienty\;=\;\frac{total\;GPE\;-\;unbond\;GPE}{total\;GPE}\;\times \;100$$

$$Loading\;efficienty\;=\;\frac{total\;GPE\;-\;unbond\;GPE}{nanoparticles\;weight}\;\times \;100$$

where *total GPE* represents the total phenols in the extract, *unbond GPE* refers to the total phenols in the supernatant, and *nanoparticles weight* refers to the weight of the zein nanoparticles (Np).

#### 2.4.4. Morphology and Size Characterization

A field emission scanning electron microscope, JSM-7800 F (JEOL, Pleasanton, CA, USA), was used for analysis. Before deposition onto an aluminum film, the samples were diluted at a 1:10 ratio. The particle size deviation was analyzed using ImageJ software (version 1.58t) based on measurements from a population of 150 particles.

#### 2.4.5. Hydrodynamic Size Determination and ζ-Potential Measurements

The size distribution and ζ-potential of ZNp, ZNp-GPE1, ZNp-GPE2, and ZNp-GPE3 samples were determined by DLS using a Zetasizer Nano ZS (Malvern Instruments, Malvern, Worcestershire, UK), with a laser of wavelength = 633 nm (He–Ne, 4.0 mW) [8].

#### 2.4.6. Spectrometer Setup for FTIR Analysis

The samples were first frozen at −40 °C using a freezer and then lyophilized. The resulting material was used for FTIR analysis. FTIR spectra were collected using a Spectrum Two FTIR Spectrometer (Perkin Elmer Inc., Waltham, MA, USA) equipped with a single attenuated total reflectance (ATR) diamond. The spectra were recorded in the range of 400–4000 cm^−1^. The collected data were processed by normalizing and graphing the spectra using OriginLab 2024 software. No corrections or modifications were applied to the spectra during the data processing.

#### 2.4.7. Experimental Design and Statistical Analysis

A completely randomized experimental design was utilized, comprising each of the treatments (ZNp-GPE1, ZNp-GPE2, and ZNp-GPE3), as well as ZNp and GPE in solution as control. We analyzed the differences between each concentration within each treatment. The experimental data were analyzed employing one-way analysis of variance (ANOVA) employing NCSS 97 software (NCSS, Inc., Kaysville, UT, USA) at a significance level of *p* = 0.05. The means analysis was carried out through a multivariate range Tukey test (Tukey post hoc test) at a 95% confidence interval. All results are presented as the mean ± standard deviation.

## 3. Results and Discussion

### 3.1. Analysis of Spectral Characteristics in the UV–Vis Range

**Figure 2:**
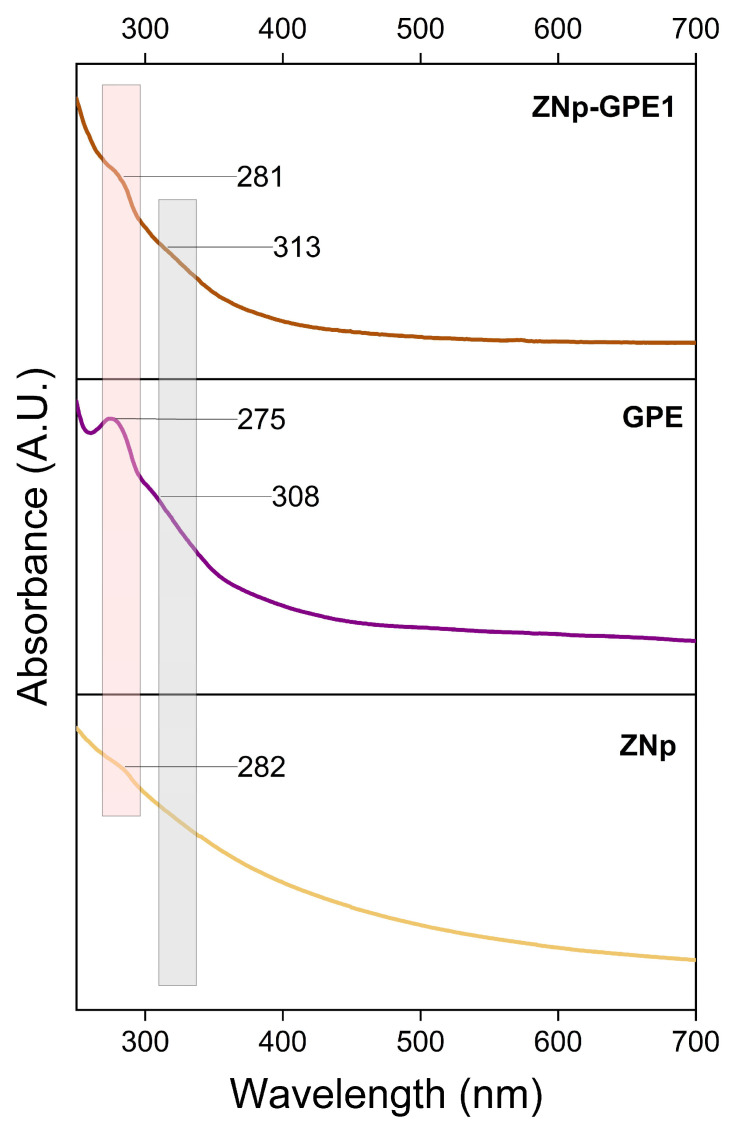
UV–vis absorption spectra of the samples: ZNp (zein nanoparticles), GPE (grape pomace extract), and ZNp-GPE1 (grape pomace extract encapsulated in zein at a 10% (*v*/*v*) concentration).

The absorption spectrum of ZNp, shown in Figure 2, exhibits a noticeable absorption band near 282 nm, which is related to the chemical structure of zein, specifically to the functional groups present in its protein chain. This value is consistent with previous studies that report the absorption of zein in this region of the UV spectrum, related to the electronic interactions of peptide bonds [35,36].

Regarding the spectrum of GPE shown in Figure 2, a prominent band is observed at 275 nm, attributed to the presence of phenolic compounds and flavonoids, which are known to absorb within the 275–310 nm range. Additionally, the spectrum shows a small shoulder around 308 nm, indicating the presence of other bioactive compounds, such as phenolic acids. These results suggest that the extraction process did not compromise the integrity of the biomolecules in the GPE, as evidenced by the observed characteristic bands of the functional groups that were expected to be extracted. Admittedly, the extraction was effective, as it adequately preserved the compounds present in the plant material, indicating the positive efficiency of the extraction process [37,38].

The spectrum shown in Figure 2 for sample ZNp-GPE1 displays a band at 281 nm, indicating the presence of functional groups from the zein, with a very slight blue shift compared to the ZNp spectrum. This suggests an interaction between zein compounds and the encapsulated GPE, resulting in changes in absorption. Additionally, a small shoulder appears at 313 nm, which may be due to the incorporation of phenolic compounds from GPE within the ZNp structure. These changes in the observed spectrum reflect the alteration in the absorption properties resulting from the encapsulation process.

UV–vis analysis of the GPE-loaded zein nanoparticles indicated that the extract was effectively incorporated, as evidenced by the characteristic absorbance peaks of the bioactive compounds present in the GPE. The absorption spectra revealed that the extract retained its spectral features, suggesting that the bioactive compounds were incorporated into the nanoparticle matrix, thereby ensuring stable inclusion in the nanoparticles.

### 3.2. Quantification of Total Phenol Content of GPE and ZNp

The quantification of total phenols in the ethanolic (50%) GPE revealed a concentration of 16.30 µmol GAE/g dw. In contrast, zein nanoparticles containing GPE (ZNp-GPE1, ZNp-GPE2, and ZNp-GPE3) exhibited significantly lower values, ranging from 2.31 to 1.94 µmol GAE/g dw (Table 1). No statistically significant differences were observed between the total phenol content of ZNp (2.80 ± 0.69 µmol GAE/g dw) and the different treatments of zein nanoparticles loaded with various GPE concentrations (*p* > 0.05), as shown in Table 1. These results suggest that the polyphenols of GPE possibly interacted with the zein matrix, suggesting a hydrophobic interaction. These findings have been reported previously [13,39].

Furthermore, ZNp exhibited a total phenol content of 2.80 ± 0.69 µmol GAE/g dw, which was higher than that of the GPE-loaded nanoparticles containing 10%, 5%, and 2.5% extract, with values of 1.94 ± 0.19, 2.29 ± 0.27, and 2.31 ± 0.17 µmol GAE/g dw, respectively (Table 1). This behavior is consistent with findings reported by Hernández-Espinosa (2015) [40], where it is established that zein, as a prolamin protein, contains a high proportion of proline, an aromatic amino acid detectable by the Folin–Ciocalteu reagent in the quantification method [41,42]. Other studies have reported higher total phenol content in Malbec GPE, with values of 19.6 and 19.5 mg GAE/g when using pure ethanol and 50% ethanol as solvents, respectively, and employing ultrasonic-assisted extraction [43]. Additionally, another study reported total phenol contents ranging from 50 to 75 µmol GAE/g dw in eight different GPE treatments using ultrasound-assisted extraction. Caldas et al. (2018) [44] reported total phenol content in GPE ranging from 45 to 48.6 mg GAE/g dw (approximately 7.65 µmol GAE/g dw) when using 50% ethanol extraction at a 1:10 solute-to-solvent ratio under conditions of 200 rpm and 30 °C. This value is lower than those found in the present study, likely due to the higher temperature and higher stirring speed. This suggests that the agitation provided by the orbital shaker may not have been sufficient to “renew” the solution in contact with the grape skin surface when larger volumes were used, thereby reducing the concentration gradient and negatively affecting mass transfer. These findings confirm the influence of extraction methods on phenolic content in grape pomace.

### 3.3. Nanoparticles Entrapment Capacity

The evaluation of the entrapment capacity of GPE in zein nanoparticles is presented in terms of association efficiency (AE) and loading efficiency (LE), as shown in Table 2. The values of AE fluctuate between 89% and 97%, and the LE values fluctuate between 4.71% and 1.08%, as shown in Table 2. The zein nanoparticles (ZnP) loaded with 10%, 5%, and 2.5% concentrations of GPE exhibited statistically significant differences in association efficiency (AE) and loading efficiency (LE). Specifically, the AE and LE values were directly proportional to the concentration of GPE loaded onto the ZnP. Studies reported in the literature [45] have shown AE and LE values exceeding 70%, similar to those observed in this study. This similarity suggests that increasing the concentration of extract or compound enhances the association rate with the nanoparticles [46].

Moreover, contrary to the findings of Wang et al. (2019) [47], who reported a carrier saturation charge of 10% for curcumin, our research demonstrated an association efficiency pattern for AE ranging from 89.56% to 97.62% for GPE concentrations between 2.5% and 10%.

The results obtained in this study indicate that ZNp-GPE has significant potential for biomedical applications, particularly in the controlled release of antioxidant compounds. In terms of encapsulation efficiency, a range of 89–97% was achieved, which is notably high compared to other studies in the field. For instance, a study by Peipei Zhu et al. (2021) reported an encapsulation efficiency of more than 90% for resveratrol-loaded zein–polyglycerol nanoemulsions [48]. However, our results not only align with this high efficiency but also demonstrate the robust capability of zein nanoparticles to protect and efficiently encapsulate bioactive compounds such as those in grape pomace extract (GPE), suggesting that zein nanoparticles could serve as a superior delivery system for various bioactive compounds with potential biomedical applications.

### 3.4. Hydrodynamic Size Determination and ζ-Potential Measurements

Nanoparticles exhibit unique physical and chemical properties that significantly influence their biomedical applications [49]. Key factors include size, surface area, shape, and surface properties, which collectively determine their interaction with biological systems [50]. In Table 3, the hydrodynamic diameter of zein nanoparticles (ZNp) is reported as 211.93 ± 2.77 nm, with a PDI of 0.0256 ± 0.015. These values are comparable to those obtained in one of our recent studies, where the hydrodynamic diameter was found to be 159.26 ± 5.96 nm [8]. Notably, nanoparticles incorporating different concentrations of GPE exhibited significantly smaller hydrodynamic diameters, with a statistically significant difference compared to ZNp (*p* < 0.05). Regarding surface charge, the measured zeta potential ranged from 11.62 ± 1.73 mV to 15.7 ± 0.30 mV, showing significant differences relative to ZNp (*p* < 0.05). These values are considerably lower than those reported in studies involving avocado extract-loaded ZNp, where the hydrodynamic size varied from 166.9 ± 14.1 nm to 305.2 ± 16.2 nm, with a PDI ranging from 0.19 to 0.26 and zeta potential values between 59.5 ± 8.1 mV and 32.9 ± 0.4 mV, as measured by DLS [51]. The lower zeta potential observed in our study suggests a reduced electrostatic repulsion between particles, which may impact colloidal stability.

Nanoparticles produced using different fabrication methods, such as nanoprecipitation, high-speed homogenization, and ultrasonic homogenization, exhibit variations in particle size due to differences in processing conditions. For example, chestnut shell extract-based nanoparticles synthesized using these techniques exhibited particle sizes ranging from 143.47 nm to 187.23 nm, 169.37 nm to 254.75 nm, and 156.57 nm to 232.45 nm, respectively [52]. Zeta potential is a key parameter in assessing the stability of colloidal dispersions. Particles with zeta potential values exceeding ±30 mV exhibit strong electrostatic repulsion, leading to enhanced physical stability in suspension [53]. The lower zeta potential observed in our study suggests moderate stability, which may lead to some degree of aggregation over time.

The PDI is critical for determining the homogeneity of nanoparticle suspensions or emulsions. Its values range from 0.0 to 1.0, where values approaching 1.0 indicate a polydisperse system with a broad size distribution, while values near 0.0 represent a monodisperse system. Higher PDI values are associated with increased aggregation tendencies due to heterogeneous particle sizes [54]. For chestnut shell extract-loaded ZNp, the PDI varied depending on the synthesis method: nanoprecipitation (0.078–0.265), high-speed homogenization (0.064–0.262), and ultrasonic homogenization (0.078–0.234). The differences between these methods were relatively small [52]. Similarly, Proenca et al. (2024) [55] reported PDI values of 0.153 ± 0.03 and 0.159 ± 0.02 for zein nanoparticles and zein nanoparticles loaded with curcumin and carvacrol, respectively. It is noteworthy that all reported values remain below 0.2, which is lower than typical PDI values for polymer-based nanoparticles synthesized using natural matrices such as zein. This suggests that the nanoparticles in this study exhibit a relatively uniform size distribution, which may enhance their stability and performance.

While various critical process variables influence diameter, PDI, and zeta potential in all synthesis methods, the amount of extract loaded emerged as a key factor in this study. ZNp resulted in smaller PDI values, indicating a more uniform particle distribution. In contrast, increasing the extract ratio showed no statistically significant difference in diameter and PDI values. These findings highlight the importance of optimizing formulation parameters to achieve desired nanoparticle characteristics for specific applications.

The hydrodynamic diameter distribution and ζ-potential of the ZNp, ZNp-GPE1, ZNp-GPE2, and ZNp-GPE3 samples were determined by DLS using a Zetasizer Nano ZS (Malvern Instruments, Malvern, Worcestershire, UK), with a laser of wavelength = 633 nm (He–Ne, 4.0 mW) [8]. DLS measurements revealed a reduction in the average hydrodynamic diameter of the nanoparticles upon GPE incorporation, with hydrodynamic diameters of 136 nm, 142 nm, and 138 nm for the 10%, 5%, and 2.5% GPE concentrations, respectively. This reduction suggests that the GPE extract may influence the size of the nanoparticles by stabilizing the surface, potentially preventing nanoparticle growth. The extract could act as a stabilizer by coating the surface of the nanoparticles, thereby helping to maintain their size and stability in solution. As the concentration of GPE increases, the surface of the nanoparticles could become densely covered with the bioactive compounds from the extract, leading to a more compact coating. This dense coating could prevent further aggregation and restrict the growth of the nanoparticles, contributing to the reduction in size observed with higher GPE concentrations. These effects have been observed in previous studies, where the addition of bioactive compounds led to stabilizing nanoparticle size and preventing growth through surface coating [26,51,56].

The ζ-potential measurements indicated that the incorporation of GPE influenced the surface charge of the nanoparticles, contributing to their stability in solution. Initially, with lower concentrations of GPE (2.5%), the surface charge increased, which suggests that the extract molecules interact with the surface of the nanoparticles, leading to increased electrostatic repulsion between particles. This behavior enhances the colloidal stability of the nanoparticles. Similar trends have been reported in the literature, where the interaction of surface-active agents or bioactive compounds with nanoparticle surfaces increases electrostatic repulsion and improves stability [26,51].

While the ζ-potential decreased with the addition of 5 and 10% GPE, the magnitude of this reduction was small, and the values remained significantly higher than those measured for zein nanoparticles without GPE. This can be explained by the fact that at higher concentrations of the extract, the surface of the nanoparticles may become densely covered with bioactive compounds from GPE, resulting in a more compact layer. The dense coating likely interfered with electrostatic repulsion, leading to the observed partial decrease in the ζ-potential. Furthermore, as the extract concentration increases, the interactions between the extract and zein may become less effective in maintaining the surface charge, potentially due to saturation or aggregation of extract molecules on the nanoparticle surface. The smaller particle sizes associated with higher GPE concentrations may further influence the surface charge distribution, reducing ζ-potential despite the increased surface area. This phenomenon has been previously described in studies where higher concentrations of bioactive compounds led to a decrease in ζ-potential, possibly due to saturation of surface binding sites and reduced electrostatic repulsion [56].

### 3.5. Morphology and Size Characterization

**Figure 3:**
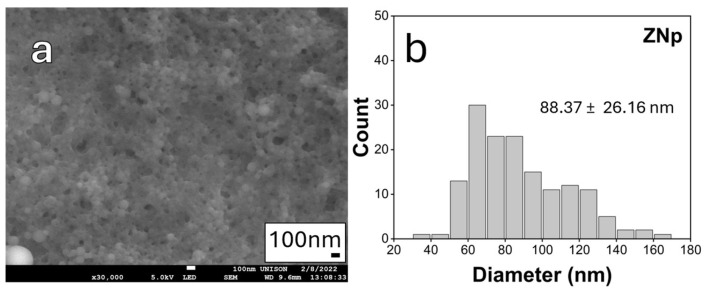
(**a**) SEM micrograph with a scale bar of 100 nm and (**b**) size distribution histogram of ZNp. Size distribution was analyzed using ImageJ software version 1.54g based on a population of 150 particles.

Analysis of SEM micrographs of the unloaded ZNp sample reveals a spherical morphology, accompanied by noticeable particle coalescence in the micrographs. This observation is consistent with previous studies that employed a similar method of synthesis [8,26,57,58,59]. The average particle size was 88.37 nm, with a relatively broad size deviation of ±26 nm, as shown in Figure 3. This size range is suitable for potential biomedical applications, such as cellular internalization [59,60].

**Figure 4:**
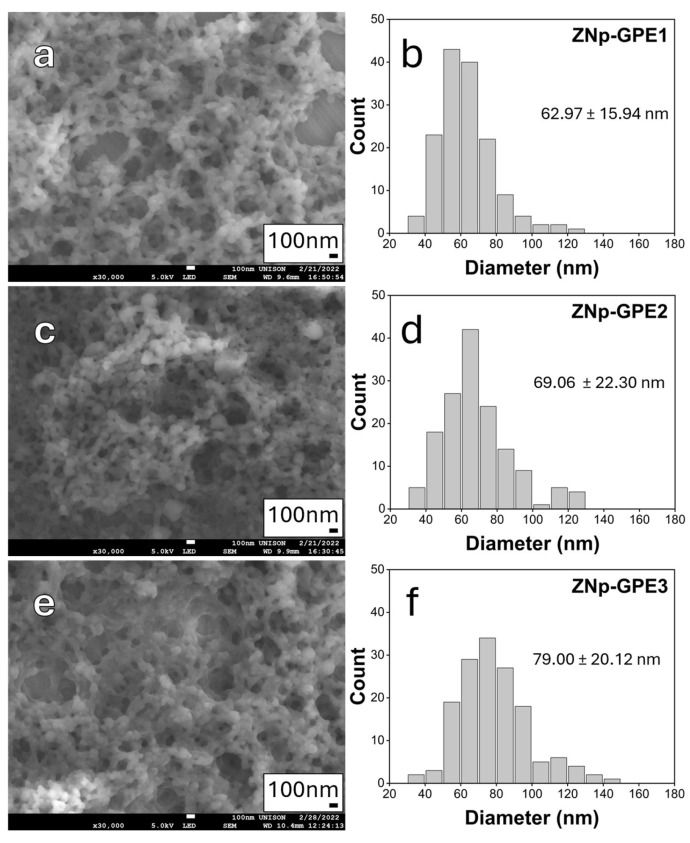
(**a**,**c**,**e**) SEM micrographs with scale bar of 100 nm, and (**b**,**d**,**f**) size distribution histograms of ZNp nanoparticles loaded with GPE: ZNp-GPE1 (**a**,**b**), ZNp-GPE2 (**c**,**d**), and ZNp-GPE3 (**e**,**f**), GPE concentrations are 10% *v*/*v*, 5% *v*/*v*, and 2.5% *v*/*v*, respectively. Size distributions were analyzed using ImageJ software based on a population of 150 particles per sample.

SEM micrographs of the samples loaded with GPE are shown in Figure 4, indicating that the incorporation of GPE did not significantly alter the spherical morphology of the nanoparticles. While a trend towards smaller nanoparticle sizes was observed with increasing GPE concentration, the differences in size distribution are not statistically significant compared to the unloaded ZNp sample. Nonetheless, this apparent reduction in nanoparticle size, with average sizes of 79 nm, 69 nm, and 62 nm for samples ZNp-GPE3, ZNp-GPE2, and ZNp-GPE1, respectively, is consistent with the results from the hydrodynamic size determination, which did statistically reveal that the unloaded ZNp sample has a larger hydrodynamic diameter than the samples loaded with GPE. This suggests that nanoparticle size may be related to loading GPE into the ZNp, providing some degree of stabilization by reducing nanoparticle size. It is essential to note that DLS size measurements are typically larger than those obtained using SEM. This is expected because DLS measures the nanoparticle, including the surrounding solvent layer, whereas SEM micrographs interact with dry nanoparticles in a vacuum. Moreover, despite these size differences, the trend of decreasing size with increased GPE concentrations is consistent between both techniques.

In addition to the trend in average nanoparticle size, the size distribution also appears to be susceptible to the GPE concentration. As shown in Table 4, the samples loaded with GPE display narrower size distributions compared to the unloaded ZNp sample, indicating an improvement in size uniformity that may also be related to the stability provided by the GPE. However, agglomeration is observed in sample ZNp-GPE1, which contains the highest concentration of GPE. This suggests a potential correlation between higher GPE concentrations and the agglomeration of loaded nanoparticles, further highlighting the importance of optimizing the concentration of GPE to balance smaller particle size and size distribution against the potential for increased agglomeration.

SEM images confirmed that the zein nanoparticles retained their spherical morphology even after incorporating GPE. A decrease in particle size was observed with GPE loading, suggesting that the extract may contribute to the structure of the nanoparticles, potentially affecting both the surface and internal composition.

Additionally, the average size of the ZNp-GPE nanoparticles was significantly smaller than that of the unloaded zein nanoparticles, indicating that the incorporation of GPE affects the physicochemical properties of the nanoparticles. The average sizes of the loaded nanoparticles were 62 nm, 69 nm, and 79 nm for the 10%, 5%, and 2.5% GPE concentrations, respectively. This behavior is consistent with previous studies, such as that of Priyanka Shinde et al. (2020), who observed a decrease in the size of zein nanoparticles when loaded with carvacrol [61]. Our results suggest that the GPE extract influences the formation and compaction of the nanoparticles.

### 3.6. Fourier-Transform Infrared Spectroscopy (FTIR) Analysis

**Figure 5:**
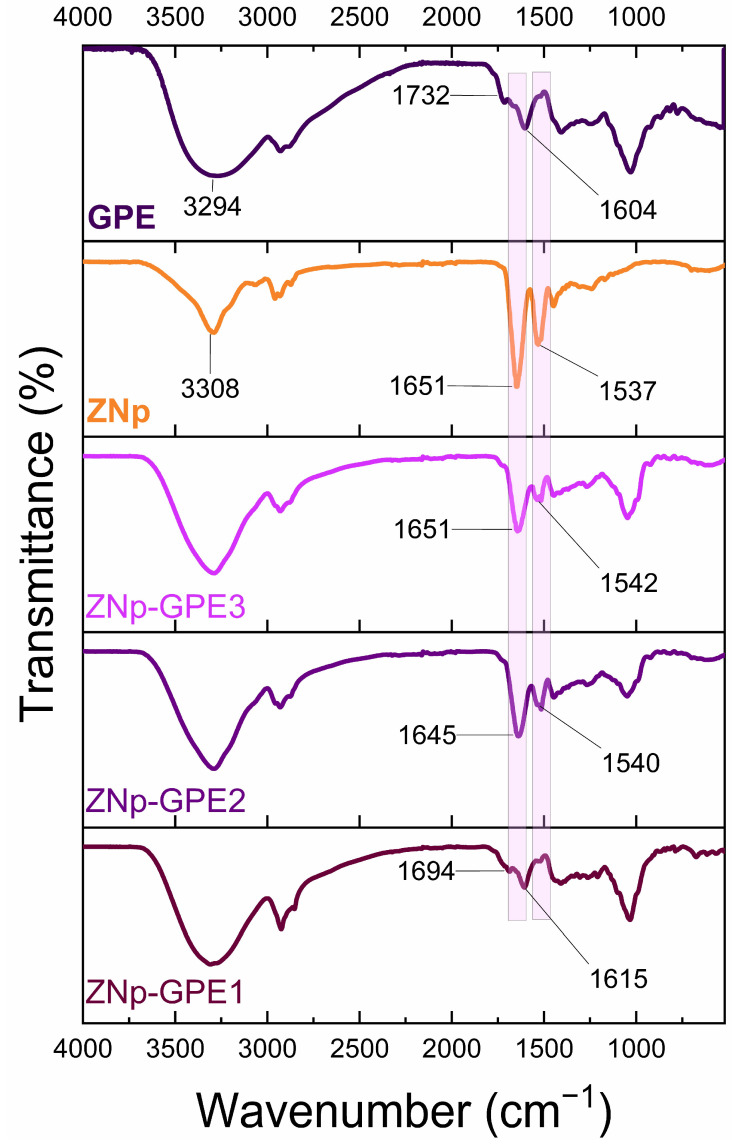
FTIR spectra of GPE (grape pomace extract), ZNp (zein nanoparticles), and ZNp-GPE formulations with different concentrations of encapsulated extract: ZNp-GPE1 (10% *v*/*v*), ZNp-GPE2 (5% *v*/*v*), and ZNp-GPE3 (2.5% *v*/*v*).

Fourier-transform infrared (FTIR) spectroscopy was employed to analyze the molecular interactions between zein nanoparticles (ZNp) and grape pomace extract (GPE). The FTIR spectrum of GPE exhibited a broad absorption peak at approximately 3294 cm^−1^, corresponding to O–H stretching vibrations, characteristic of hydroxyl groups in alcohols and phenolic compounds (Figure 5). Additionally, the spectral band at 1604 cm^−1^ was attributed to C=O stretching in ester groups, indicative of the presence of fatty acids, pectins, lignins, or tannins [6].

In the case of ZNp, the FTIR spectrum showed an absorption peak at 3308 cm^−1^, corresponding to O–H stretching due to hydrogen bonding. The characteristic peaks at 1651 cm^−1^ and 1537 cm^−1^ corresponded to amide I (C=O stretching) and amide II (C–N stretching and N–H bending) regions, respectively [62].

The interaction of polyphenols extracted from GPE with ZNp can be confirmed by the shifts in the amide I and II bands, suggesting interactions between the extract and the zein matrix. These interactions could occur both on the surface of the nanoparticles and within the nanoparticle matrix. The shifts in the amide bands indicate that the functional groups of the GPE may bind to or interact with the functional groups of zein, suggesting a complex incorporation mechanism. These findings are consistent with previous studies such as Xing et al. [13], Wei et al. [63], Ahmad and Gani [64], and Mohsen et al. [65], which observed similar FTIR spectral modifications when encapsulating pomegranate peel extract into zein nanoparticles.

Furthermore, Moreno et al. (2019) [66] demonstrated that zein fibers loaded with phenolic-enriched orange extracts exhibited FTIR spectral shifts in the amide regions, confirming chemical interactions between zein and polyphenols. Similarly, López de Dicastillo et al. (2019) [67] reported FTIR spectral changes indicative of specific interactions between zein and bioactive compounds in açaí fruit extract-loaded nanoparticles.

Collectively, these results emphasize the utility of FTIR spectroscopy in confirming the formation of zein–polyphenol conjugates. The observed spectral modifications suggest the presence of intermolecular interactions, likely enhancing the antioxidant properties of the nanoparticles, with promising applications in the food and pharmaceutical industries.

## 4. Conclusions

This study demonstrates that zein nanoparticles loaded with GPE have high potential in biomedical applications, mainly due to their ability to encapsulate bioactive compounds with antioxidant properties. The high encapsulation efficiency (89–97%) suggests that zein nanoparticles can effectively protect the phenolic compounds from GPE, improving their stability against environmental factors such as light and oxygen. In addition, utilizing the by-products of industries such as wine production provides a sustainable solution by extracting compounds that can be used in a wide range of applications, thereby generating natural and environmentally friendly alternatives. The results indicate that nanoparticles incorporating different concentrations of GPE exhibited significantly smaller hydrodynamic diameters, with a statistically significant difference compared to ZNp. SEM analysis further confirmed the size of the nanoparticles with a spherical shape and improvements in their size deviation when loaded with GPE. The quantification of total phenols revealed a significant reduction in phenolic content after encapsulation, suggesting strong interactions between the GPE and the zein matrix. The analysis of infrared spectroscopy also supported these interactions. Overall, this study highlights the feasibility of using zein nanoparticles for encapsulating fruit-derived polyphenols, with potential applications in both the pharmaceutical and food industries. The observed chemical interactions may enhance the functional properties of the nanoparticles, particularly their antioxidant capacity, thereby paving the way for improved formulations in nutraceutical and therapeutic applications.

## Figures and Tables

**Table 1:** Total phenol content in GPE, ZNp, and ZNp loaded with GPE.

Sample	Total Phenols µmol GAE/g dw
GPE	16.30 ± 0.88 ^a^
ZNp	2.80 ± 0.69 ^b^
ZNp-GPE1	1.94 ± 0.19 ^b^
ZNp-GPE2	2.29 ± 0.27 ^b^
ZNp-GPE3	2.31 ± 0.17 ^b^

ZNp: zein nanoparticles. GPE: grape pomace extract. ZNp-GPE1: 10%; ZNp-GPE2: 5%; ZNp-GPE3: 2.5% (*v*/*v*). Values are an average of three measurements. Values are an average of three measurements. Data, along with standard errors, represent the means of at least three experiments. Treatment means were separated using the Tukey test (*p* < 0.05). a, and b: different letters in superscript indicate significant differences in the column (*p* < 0.05).

**Table 2:** Association and loading efficiency of ZNp-GPE treatments.

Sample	AE (%)	LE (%)
ZNp-GPE1	97.62 ± 0.029 ^a^	4.71 ± 0.001 ^a^
ZNp-GPE2	94.99 ± 0.120 ^b^	2.29 ± 0.003 ^b^
ZNp-GPE3	89.56 ± 0.169 ^c^	1.08 ± 0.002 ^c^

ZNp: zein nanoparticles. GPE: grape pomace extract. ZNp-GPE1: 10%; ZNp-GPE2: 5%; ZNp-GPE3: 2.5% (*v*/*v*). Values are an average of three measurements. Values are an average of three measurements. Data, along with standard errors, represent the means of at least three experiments. Treatment means were separated using the Tukey test (*p* < 0.05). a, b, and c: different letters in superscript indicate significant differences in the column (*p* < 0.05).

**Table 3:** Mean hydrodynamic diameter, polydispersity index, and ζ-potential values.

Sample	Hydrodynamic Diameter	PDI	ζ-Potential (mV)
ZNp	211.93 ± 2.77 ^b^	0.0256 ± 0.015 ^c^	7.28 ± 0.49 ^c^
ZNp-GPE1	136.3 ± 3.31 ^a^	0.115 ± 0.013 ^a^	11.62 ± 1.73 ^a^
ZNp-GPE2	142.3 ± 2.31 ^a^	0.148 ± 0.015 ^a^	13.4 ± 0.55 ^ab^
ZNp-GPE3	138.2 ± 1.49 ^a^	0.193 ± 0.015 ^b^	15.7 ± 0.30 ^b^

ZNp: zein nanoparticles. GPE: grape pomace extract. ZNp-GPE1: 10%; ZNp-GPE2: 5%; ZNp-GPE3: 2.5% (*v*/*v*). Values are an average of three measurements. Data, along with standard errors, represent the means of at least three experiments. Treatment means were separated using the Tukey test (*p* < 0.05). a, b, and c: different letters in superscript indicate significant differences in the column (*p* < 0.05).

**Table 4:** Average size and size standard deviation.

Sample	Average Size (nm)
ZNp	88.37 ± 26.16 ^a^
ZNp-GPE1	62.97 ± 15.94 ^a^
ZNp-GPE2	69.06 ± 22.30 ^a^
ZNp-GPE3	79.00 ± 20.12 ^a^

ZNp: zein nanoparticles. ZNp-GPE1: 10%; ZNp-GPE2: 5%; ZNp-GPE3: 2.5% (*v*/*v*). Particle size and size standard deviation were analyzed using ImageJ software based on a population of 150 particles per sample. Values are presented as mean ± standard error. Treatment means were separated using the Tukey test (*p* < 0.05). a in superscript indicate significant differences in the column (*p* < 0.05).

## Data Availability

The data presented in this study are available on request from the corresponding author.

## Abbreviations

The following abbreviations are used in this manuscript:

GPE	Grape pomace extract
SEM	Scanning electron microscopy
FTIR	Fourier transform infrared spectroscopy
DLS	Dynamic light scattering
PDI	Polydispersity index
